# Novel *EGFR* V834L Germline Mutation Associated With Familial Lung Adenocarcinoma

**DOI:** 10.1200/PO.17.00266

**Published:** 2018-07-16

**Authors:** Cor van der Leest, Anja Wagner, Rute M. Pedrosa, Joachim G. Aerts, Winand N.M. Dinjens, Hendrikus J. Dubbink

**Affiliations:** **Cor van der Leest** and **Joachim G. Aerts,** Amphia Ziekenhuis, Breda; and **Anja Wagner, Rute M. Pedrosa, Joachim G. Aerts, Winand N.M. Dinjens,** and **Hendrikus J. Dubbink,** Erasmus University Medical Center, Rotterdam, Netherlands.

## INTRODUCTION

Lung cancer is a worldwide, known disease with a high mortality rate and is often related to nicotine abuse. Family-related lung cancer has been reported occasionally, but little is known about the genetic backgrounds. With routine analysis for targetable mutations, unexpected potentially pathogenic mutations may be found. Here, we present a case study of a patient with a targetable somatic *EGFR* mutation and a novel *EGFR* germline variant, which also occurred in other family members with lung cancer.

## INDEX PATIENT

**Fig 1.**
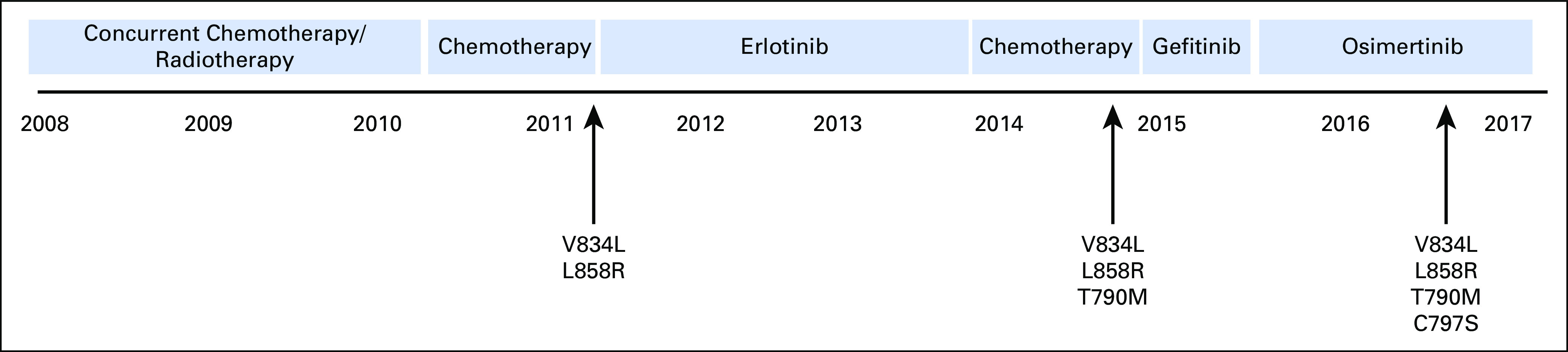
Timeline of treatment and detection of acquired somatic *EGFR* mutations of the index patient. The germline *EGFR* variant V834L is indicated at all time points.

Our female index patient of Surinam origin, a former smoker with a 10 pack-year history, was diagnosed at the age of 57 years with non–small-cell lung cancer (NSCLC), stage IIIA cT2bN2M0 adenocarcinoma. Initial treatment included concurrent chemotherapy and radiotherapy, and palliative chemotherapy after recurrence (the treatment timeline is shown in Fig 1). After a second recurrence, a new biopsy of a lymph node metastasis was obtained for *EGFR* mutation analysis by next-generation sequencing. This analysis revealed a well-known oncogenic L858R mutation along with a V834L variant, which are both *EGFR* exon 21 substitutions and are present in the same allele (in *cis*). The V834L variant seemed to be present in the germline, because both normal adjacent lymph node tissue and lymphocyte-derived DNA from whole blood of the index patient showed this variant.

The patient was treated with the EGFR tyrosine kinase inhibitor (TKI) erlotinib. At the fourth progression of the disease, the patient was retreated with chemotherapy. After the fifth progression of the disease, a new biopsy revealed a first-generation TKI-resistance *EGFR* T790M mutation, in addition to L858R and V834L. Therefore, treatment was switched to the third-generation EGFR TKI osimertinib. Because of progression after 1 year of osimertinib treatment, a liquid biopsy was taken and cell-free DNA was isolated. Subsequent mutation analysis showed that the progression was probably because of the development of *EGFR* resistance mutation C797S in *cis* with T790M (data not shown). Performance of the patient was declining, and there were no remaining therapeutic options. Best supportive care was given, and the patient eventually died.

## PATIENT’S FAMILY HISTORY WAS POSITIVE FOR LUNG CANCER

The patient’s younger brother, who was 57 years old and a current smoker, was diagnosed in 2016 with stage IV, *EGFR* L858R mutation–positive NSCLC. He was treated with erlotinib, and his disease progressed after 3 months. A new biopsy was obtained, revealing no targetable secondary resistance mechanism, such as T790M mutation or *MET* amplification. Standard chemotherapy was started, but the patient died because of disease progression and decline in performance status.

A sister and daughter of our index patient, 46 and 42 years old, respectively, both nonsmokers, were diagnosed with stage IV NSCLC before the knowledge and treatment options of *EGFR* mutations. They were both treated with palliative chemotherapy but died as a result of disease progression. The patient’s father died at a young age as the result of massive hemoptysis, without a diagnosis.

After written consent and permission were obtained from the index patient and family members, *EGFR* mutation and germline variation analyses were performed on tumor samples and healthy tissue, respectively, of the three affected relatives. Because our study started after the deaths of the index patient’s brother, sister, and daughter, no blood was available; only archival formalin-fixed paraffin-embedded normal and tumor tissues were available.

Like our index patient, all three affected family members had a heterozygous *EGFR* V834L germline variant in normal tissue and the same somatic *EGFR* L858R mutation in *cis* with the V834L variant in their respective tumor tissues. No tissue samples from the patient’s father were available.

**Fig 2.**
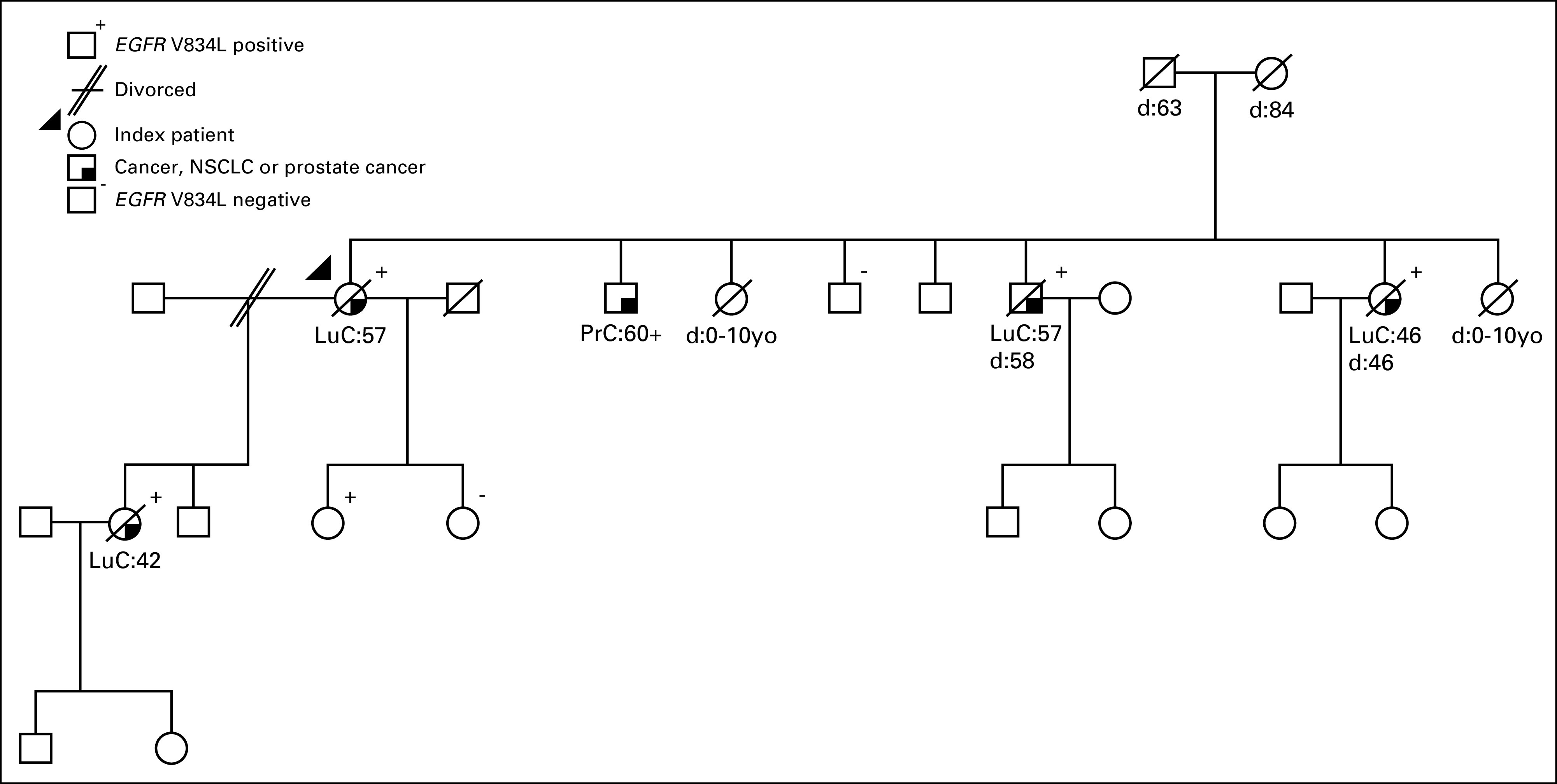
Pedigree of a family with non–small-cell lung cancer (NSCLC) associated with the presence of an *EGFR* V834L germline variant. Squares and circles represent male and female family members, respectively. Diagonal strikes represent deceased family members. Age at diagnosis of the indicated cancer or at death (d) is indicated below the squares and circles. LuC, lung cancer; PrC, prostate cancer; yo, years old.

Further germline research was performed in four healthy family members who did not have signs of lung cancer. One of the index patient’s children, who was 36 years old, showed the same heterozygous *EGFR* V834L germline variant. All other investigated family members were wild type at this position in *EGFR* (Fig 2).

## DISCUSSION

Little is known about hereditary lung cancer. This is probably in part because lung cancer is strongly associated with smoking and therefore a familial association may be easily missed. To our knowledge, this is the first report of a family with multiple cases of NSCLC associated with germline transmission of an *EGFR* V834L germline variant; all tumors in the four family members additionally harbored a somatic *EGFR* L858R mutation in *cis*. All except one of the known V834L carriers developed lung cancer during their lifespan, but so far none of the investigated noncarriers.

There are only a few reports documenting families with proven inherited *EGFR* variants over generations that probably conferred predisposition to lung cancer.^1,2^ In a family with *EGFR* V843I mutations, four family members in two generations (three of them with proven germline variants) developed lung adenocarcinoma.^1^ As in the family we studied, all of these adenocarcinomas displayed a somatically-acquired L858R mutation in *cis* with V843I. The index patient in the family with *EGFR* V843I mutations was treated with erlotinib and gefitinib, but without response, even though the L858R mutation was present. Also, in vitro, the V843I substitution conferred TKI resistance. Yu et al^2^ described a family carrying a germline T790M mutation. In their study, both the index patient and her mother developed lung adenocarcinomas. Interestingly, multiple distinct synchronous tumors of the index patient displayed different additional *EGFR* mutations (ie, deletions of 3 and 15 bp in exon 19 and L858R).^2^ A report by Bell et al^3^ describes a family with five members affected by lung cancer. Proven germline *EGFR* T790M mutations were present in three patients in two generations.^3^ In distinct lesions in two of these patients, the mutation co-occurred with different *EGFR* substitutions (L858R, L747-T751del, and G716A), all in *cis* with T790M. Because T790M is a well-known TKI resistance mutation, no response was observed upon gefitinib treatment in the only TKI-treated patient of this family, as expected.

Incidental cases of germline *EGFR* variants in patients with lung cancer have been reported. These have included variants such as V769M, R776H, and T790M substitutions in exon 20 and R831C, V843I, and P848L substitutions in exon 21.^4-9^ However, in the absence of family histories, it is unclear whether these variants predisposed these patients to lung cancer. Importantly, as described above, these germline variants often co-occur in the tumor with other, mostly well-known, actionable *EGFR* mutations. This strongly indicates that somatically acquired secondary *EGFR* mutations are indispensable for tumorigenesis.

We hypothesize that presence of the *EGFR* V834L germline variant is causative for the high incidence and early onset of lung cancer in the family we studied. So far, all lung cancers in this family have been found to harbor both the *EGFR* V834L variant and the well-described oncogenic *EGFR* L858R mutation. Although this is a relatively small cohort, this may indicate that the V834L variant is not or only weakly oncogenic and by itself is not sufficient to drive uncontrolled growth. However, when a second oncogenic mutation in *cis* is present, cell growth accelerates more than proportionally.

This hypothesis is in line with eight described *EGFR* V834L variants found in the Catalogue Of Somatic Mutations In Cancer database.^9^ The V834L variant in the lung tumors of all of these patients, with unknown germline status, is accompanied by the L858R mutation (seven times) or by an exon 19 deletion.^10-12^ The reason for the apparent preference of V834L for L858R is unclear. Such a preference is not unique (eg, the germline variant *EGFR* V769M preferentially associates with mutations of codon G719 and/or S768). This is possibly because the energy balance of the EGFR V769M–substituted protein does not favor combination with L858R.^9^ Structural and functional analysis of the V834L variant alone and/or in combination with strong oncogenic driver mutations can provide additional mechanistic information on its pathogenicity and preferred association with other oncogenic *EGFR* mutations.

Until now, lung cancer surveillance programs have been mainly recommended for active smokers or patients with a smoking history of 30 pack-years but who have stopped within the last 15 years. Although smoking is unmistakably associated with lung cancer development, increased awareness of genetic factors that predispose individuals to lung cancer is necessary. Currently, the prevalence of germline *EGFR* T790M mutations in patients with lung cancer and their relatives is being studied in the INHERIT EGFR trial (NCT01754025), to further investigate the role of *EGFR* mutations in lung cancer development. For people with a germline *EGFR* mutation and/or a strong family history of lung cancer, clinical follow-up may be recommended.

In conclusion, we describe a novel *EGFR* V834L germline mutation illustrating that genetic factors may play an important role in lung cancer predisposition and should be evaluated to optimize surveillance and clinical genetic counseling.
